# Associations Between Trauma, Early Maladaptive Schemas, Personality Traits, and Clinical Severity in Eating Disorder Patients: A Clinical Presentation and Mediation Analysis

**DOI:** 10.3389/fpsyg.2021.661924

**Published:** 2021-03-31

**Authors:** Paolo Meneguzzo, Chiara Cazzola, Roberta Castegnaro, Francesca Buscaglia, Enrica Bucci, Anna Pillan, Alice Garolla, Elisa Bonello, Patrizia Todisco

**Affiliations:** ^1^Eating Disorders Unit, Casa di Cura “Villa Margherita”, Arcugnano, Italy; ^2^Department of Neuroscience, University of Padova, Padova, Italy

**Keywords:** eating disorders, trauma, early maladaptive schema, personality traits, anorexia nervosa, bulimia nervosa, binge eating disorder, dissociation

## Abstract

**Background**: The literature has shown a significant association between traumatic experiences and eating psychopathology, showing a greater symptomatology in patients with trauma history. Less is known about the associations between trauma and cognitive schemas, and personality traits and the differences between childhood and adulthood trauma experiences. Thus, this paper aims to assess the clinical and psychological characteristics of eating disorder (ED) patients, looking for differences between patients without a history of trauma and patients with trauma experiences, as well as at possible differences between exposure in childhood, adulthood, or repeated events. Another aim of the paper is to evaluate the possible mediation role of cognitive schemas and personality traits in the relationship between early trauma and eating psychopathology.

**Methods**: From January to November 2020, 115 consecutive inpatients admitted for a specific multidisciplinary ED treatment in a dedicated Unit were evaluated for trauma, differentiating between trauma occurring in childhood and adulthood. The subjects were evaluated for early maladaptive schemas (EMS), personality traits, trauma symptomatology, quality of life, and specific psychopathologies linked to EDs. Mediation analyses between childhood and adulthood trauma and eating psychopathology were performed, with EMS and personality traits as mediators.

**Results**: Patients with a history of trauma showed higher physical and psychological symptomatology scores, with a more impaired clinical profile in patients with both childhood and adulthood trauma exposure. The mediation analysis showed a specific mediator role for the “disconnection and rejection (DR)” EMS factor in the relationship between childhood trauma (cT) and eating psychopathology.

**Conclusion**: Trauma experiences are associated with more severe clinical symptomatology in EDs and may need a specific assessment in patients with failed outpatient standard treatments. Specific cognitive schemas linked to DR domain should be evaluated in treatments for ED patients with history of trauma due to the mediation role between trauma and eating psychopathology. The need for outcome studies about treatment approaches for ED patients with history of trauma is discussed.

## Introduction

Eating disorders (EDs) are serious mental illnesses characterized by persistent unhealthy eating behaviors, distorted beliefs, and extreme concerns about weight and shape (American Psychiatric Association, 2013; Solmi et al., 2018a). Psychological impairments have been correlated with clinical, interpersonal, and biological elements, showing the complexity of the biopsychosocial model of EDs (Troop and Treasure, 1997; Frank, 2016; Solmi et al., 2018b). Recently, a growing body of literature has underlined the possible role of traumatic life events in the development of EDs, suggesting the possible presence of a specific echophenotype subgroup of patients with a history of trauma (Monteleone et al., 2020b).

Trauma is defined by event(s), experience(s), and effect(s) – the “three E’s” (Brewerton, 2019). The literature has pointed that adverse events are experienced differently depending on personal factors including race, culture, genetic, gender, and social support, which requires specific focus during the assessment and treatment of EDs due to the presence of a vulnerability to criticism, misperception of social cues, and neuropsychological impairments (Harrison et al., 2010; McFillin et al., 2012). Many studies have documented the trauma history of patients with EDs and have shown the importance of these events in the development and maintenance of the disorders during both childhood/adolescence and adulthood (Dalle Grave et al., 1996; Backholm et al., 2013; Monteleone et al., 2020b; Scharff et al., 2021). A recent comprehensive meta-analysis reviewed the existing literature, showing that the prevalence of childhood maltreatment is higher in all the ED diagnoses relative to healthy controls and other psychiatric patients (Molendijk et al., 2017). The ED behaviors have been pointed out as a dysfunctional way to escape and avoid trauma-related emotions and thoughts, acting as a maintenance factor for both ED and trauma-related symptomatology (Trottier and MacDonald, 2017). The presence of trauma has a biological impact on the management of stress, with a dysregulation of the body stress response system and the development of dysfunctional responsive behaviors (e.g., impulsivity), which have a negative impact on treatment outcome (Corstorphine et al., 2007; Monteleone et al., 2015, 2020a). Traumatic events also have a negative impact on the ED patients’ quality of life, increasing symptomatology and requiring specific treatments (Brewerton et al., 2020). With reference to clinical presentation, childhood sexual abuse has been correlated in EDs to multi-impulsivity, with the presence of binge-purging behaviors, substance abuse, and self-harm behaviors, while childhood physical abuse was associated with underweight (Caslini et al., 2016; Trottier and MacDonald, 2017).

Moreover, the presence of an internal critical “voice” has been linked to childhood trauma (cT), showing its meaningful role in the severity of the disorder as a maintaining factor with impacts on low self-esteem and high criticism levels (Pugh et al., 2018). Fewer studies have examined the relationship between EDs and traumatic events during adulthood, showing a specific association between EDs and sexual abuse, and more recently, between EDs and lockdown experience (Dubosc et al., 2012; Trottier and MacDonald, 2017; Monteleone et al., 2021a,b). Therefore, some authors have suggested that some cases need two treatment levels: one to control harmful and compromising behaviors and a second one to change the specific psychopathology (Corstorphine et al., 2007; Castellini et al., 2018; Todisco et al., 2020). If the extended psychopathological core of ED seems to remain stable and is characterized by an overestimation of weight and shapes, depression (D), anxiety, and interpersonal ineffectiveness, patients with and without a history of trauma history seem to show different psychological phenotypes that require a different specific focus (Solmi et al., 2018b; Rodgers et al., 2019). However, more longitudinal studies are needed to evaluate the specific needs linked to patients’ traumatic personal histories to achieve higher rates of effectiveness in treatments, which are now unsatisfactory (Backholm et al., 2013; Olofsson et al., 2020; Scharff et al., 2021).

Traumatic experiences have also been included in the multifactorial causes of dysfunctional personality traits – people’s characteristic patterns of thoughts, feelings, and behaviors – and cognitive schemas, such as the early maladaptive schemas (EMS) – which are pervasive principles used for the interpretation of reality and originate from unmeet universal psychological core needs (Young et al., 2003; Jovev et al., 2004; Sansone and Sansone, 2007; Carvalho et al., 2015). Indeed, patients with traumatic histories have exhibited higher levels of personality traits and EMS than patients without a history of trauma (Unoka et al., 2010; Trottier and MacDonald, 2017; Scharff et al., 2021). Previous findings have shown that both personality traits and EMS have a role in the development of clinical profiles that are closely correlated with the emotional factors targeted in EDs treatment (Moulton et al., 2015; Trottier and MacDonald, 2017; Aloi et al., 2020; Meneguzzo et al., 2020a). Personality traits linked to multi-impulsivity behaviors like borderline, antisocial, and paranoid personality have been linked to the cascade effects of traumatic events in ED patients, with dysfunctional coping behaviors adopted in order to avoid thoughts, emotions, and memories (Sansone and Sansone, 2007). Moreover, different traits emerged as strongly linked to eating psychopathology with relevant effects on prognosis and treatment, such as: insecure attachment, high-functioning/perfectionistic trait, constricted/overcontrolled tendency, and emotional dysregulation (Westen and Harnden-Fischer, 2001; Tasca and Balfour, 2014). On the other side, specific EMSs such as disconnection and impaired autonomy have shown a robust association with specific psychopathology such as EDs, supporting the use of specific cognitive-behavioral interventions that target core beliefs (Karatzias et al., 2016). Cognitive distortion such as a lack of autonomy, emotional regulation difficulties, and interpersonal problems are pervasive elements of chronic mental illness, and early detection could positively impact recovery (Pauwels et al., 2016; Pilkington et al., 2020). For these reasons, a growing body of literature has started to report the results of the integration of elements from third-wave therapies with cognitive-behavioral treatment protocols, with encouraging results (Öst, 2008; Ben-Porath et al., 2020; Pisetsky et al., 2020; Todisco et al., 2020). However, a recent meta-analysis found that there is still a lack of understanding of the effect of trauma on the psychological features and outcomes of ED patients, showing that more longitudinal studies are needed (Molendijk et al., 2017).

Given this background, this study’s first aim was to assess whether specific psychopathology, psychosocial impairments, and quality of life in EDs patients with traumatic experiences showed worse levels than patients without a traumatic history. We aimed to determine if there were any differences between patients with trauma exposure during childhood, adulthood, or multiple traumatic life events. Based on the literature data, the second hypothesis was that traumatic exposure could affect eating psychopathology and that EMSs and personality traits could act as mediators of this effect. From this perspective, the paper also aims to evaluate the direct and indirect effects of traumatic events on eating psychopathology, looking for possible specific mediators that might represent specific therapeutic targets.

## Materials and Methods

### Clinical Sample

From January 2020 to November 2020, consecutive patients admitted to the Eating Disorders Unit of the Casa di Cura Villa Margherita (Arcugnano, Vicenza, Italy) were included in this study after being admitted for inpatient treatment. The inclusion criteria were: (a) age between 15 and 60 years; (b) no severe psychiatric (e.g., schizophrenia, schizoaffective disorder, and intellectual disability) or medical acute comorbidity, neurological trauma or disorder, or drug addiction; (c) the ability to fulfill psychometric evaluations; and (d) the sign of the informed consent by the participant (or by the parents for individuals below 18 years of age). The ED diagnoses were made according to DSM-5 criteria by fully trained psychiatrists, with a specific clinical interview before the beginning of the inpatient treatment (American Psychiatric Association, 2013).

The study was part of a clinical evaluation of ED patients hospitalized in the Unit, and it was approved by the internal revision commission; it complies with the provisions of the Declaration of Helsinki and amendments.

### Measurements

The patients completed a series of self-administered questionnaires during the 1st week after their inpatient admission. Several self-report questionnaires were included in the battery to evaluate different aspects of participants’ psychopathology.

#### General and Specific Psychopathology

General psychopathology was evaluated with the Symptom Checklist-90-Revised (SCL-90R), a 90-item self-reported inventory with a five-point Likert scale with 0 indicating “not at all” and four indicating “extremely often”; higher scores indicate higher psychopathology (Derogatis and Lazarus, 1994). Psychosocial functioning was evaluated with the Clinical Impairment Assessment Questionnaire (CIA), a 16-item self-reported questionnaire with a four-point Likert scale with a 0 indicating “not at all” and three indicating “a lot”; the obtained scores range between 0 and 48, and a higher score reflects more significant impairment (Bohn et al., 2008). The specific eating psychopathologies were evaluated with the Eating Disorder Examination Questionnaire (EDE-Q), a self-reported measure composed of 28 items rated on a Likert rating scale ranging from 0 to 6, with higher scores indicating greater eating-related psychopathology (Fairburn and Beglin, 1994). The EDE-Q generates a total score and four subscales: Restraint, Eating Concern, Shape Concern, and Weight concern. The Italian Eating Disorder Quality of Life test (IEDQOL) is a 33-item scale rated on a five-point Likert scale and was used to assess the health-related quality of life perceived, with higher scores indicating poorer quality of life (Meneguzzo et al., 2020b).

#### Trauma

The Childhood Trauma Questionnaire (CTQ) is a 25-item self-reported questionnaire with a five-point Likert scale (from “never” to “frequently”) and was used for the assessment of traumatic experiences in infancy (Innamorati et al., 2016). The total score varies from 25 (no trauma) to 125 (extreme trauma), and the presence of a significant trauma was considered when scores were moderate or severe in at least one of the subscales (emotional abuse ≥13, physical abuse ≥10, sexual abuse ≥8, emotional neglect ≥15, and physical neglect ≥10; Bernstein et al., 2003). For the evaluation of lifetime traumatic experiences, the Brief Trauma Questionnaire (BTQ) was used; this is a 10-item questionnaire used to evaluate the exposure to 10 traumatic events that were classified as a cT if they occurred before the patient was 14 years old or as an adult trauma (aT) otherwise (Schnurr et al., 1999). The BTQ score is from 0 (no trauma; nT) to 10 (traumatic experiences in each investigated category). The Trauma Symptoms Inventory (TSI) was used to evaluate the specific symptomatology linked to the trauma history; the TSI has a 4-point forced response (Briere et al., 1995). Higher scores indicate greater symptomatology linked to trauma, both for the 10 standard subscales (AA: anxious arousal; D: depression; AI: anger/irritability; IE: intrusive experiences; DA: defensive avoidance; DIS: dissociation; SC: sexual concerns; DSB: dysfunctional sexual behavior; ISR: impaired self-reference; and TRB: tension-reduction behavior) and for the three factors considered: trauma, dysphoria-self, and sex problems (Gambetti et al., 2011).

For the aim of the study, patients were asked if the trauma reported was before and/or after the adolescence using the BTQ, allowing to include the participant into a specific trauma subgroup: nT, cT, aT, and both childhood and adult trauma (bT). Both BTQ and CTQ were included in the study to evaluate trauma history and quantify the severity of childhood trauma.

#### Cognitive Schemas and Personality Traits

Early maladaptive schemas were evaluated with the short version of the Young Schema Questionnaire (YSQ-S3), a 90-item self-reported instrument used for assessing four domains: disconnection and rejection (DR), impaired autonomy and performance (IAP), excessive responsibility and standards (ERS), and impaired limits (IL; Young et al., 2005; Aloi et al., 2020). The evaluation of the personality traits was performed with the short version of the Temperament and Character Inventory (TCI-140), a 140-item questionnaire with a five-point Likert-like scale from “absolutely false” to “absolutely true,” and the results were reported using the seven subscales: novelty seeking (NS), harm avoidance (HA), reward dependence (RD), persistence (P), self-directedness (SD), cooperativeness (C), and self-transcendence (ST; Vespa et al., 2015).

### Statistical Analysis

Participants were divided into four subgroups according to the presence (trauma+) or the absence (trauma−) of traumatic experiences during their life and the period of the life that they happen (cT, aT, and bT). Different *t*-tests were applied to evaluate differences between trauma+ and trauma− subgroups regarding demographic data and self-report results, and chi-square analysis for evaluating possible differences in diagnosis distribution. Different ANOVAs were calculated to evaluate the differences between trauma+ subgroups, using *post hoc* analysis with Bonferroni correction for multiple comparisons. Results were confirmed with General Linear Model (GLM) analysis with age, BM, and diagnosis as covariates. Partial eta-squared was used for the effect size evaluation. Mediation analysis was performed using the SPSS PROCESS macro-extension (version 3.5), applying Model 4 (Hayes, 2017). For mediation analysis, according to the second aim of the study, CTQ total score and BTQ were used as independent variables in different mediation analyses. The EMS domains and TCI subscales were evaluated as mediators of specific eating disorder psychopathology (EDE-Q total score was the dependent variable). The bootstrapping sampling distributions of the indirect effects were set to 5,000, and the bias level was set to 95%. Sobel test analysis was performed as a confirmatory analysis showing the overall indirect effect of the mediation analysis. The alpha was set at *p* < 0.05 for all analyses. The entire analysis was conducted with IBM SPSS Statistics 25.0 (SPSS, Chicago, IL, United States).

## Results

### Clinical Presentation

A total sample of 115 patients was included. The sample was composed of five men and 110 women, 68 patients (59%) met the criteria for anorexia nervosa (AN), of whom 37 with a restrictive AN and 31 with a binge-purge subtype, 37 patients (32%) met the criteria for bulimia nervosa (BN), 10 subjects (9%) met the criteria for binge eating disorder (BED). Pharmacological treatment was already present at the admission of 98 patients (85.2% of the sample), and all participants failed at least one ED outpatient treatment protocol. The participants had a mean age of 26.30 ± 10.27 years (14–59) and a mean body mass index (BMI) of 18.83 ± 6.75 kg/m^2^ (11.46–50.49).

Eighty-seven patients out of 115 (75.6%) reported some significant traumatic event during their lives, but between the trauma+ and trauma− subgroups, no significant differences were found in terms of age [trauma− 23.74 ± 9.94, trauma+ 27.09 ± 10.29 years, *t*(112) = −1.489, *p* = 0.139, *d* = 0.331] and BMI [trauma− 16.74 ± 3.68, trauma+ 19.47 ± 7.34, *t*(112) = −1.857, *p* = 0.066, *d* = 0.053]. Looking at the distribution of the diagnosis, 48 out of 68 AN patient were included into the trauma+ subgroup (70.6%), 30 out of 37 BN patients were included into the trauma+ subgroup (81.1%), and all the patients with BED diagnosis. The comparison between trauma− group vs. trauma+ subgroups considered as a whole showed lower scores in all the psychopathological scales included in the study [SCL90R total: 131.26 ± 50.37 vs. 172.68 ± 59.71, *t*(110) = −3.253, *p* = 0.002, *d* = 0.750; EDE-Q total: 3.37 ± 1.32 vs. 4.36 ± 1.07, *t*(110) = −3.579, *p* = 0.001, *d* = 0.824; CIA: 28.70 ± 9.88 vs. 35.71 ± 9.48, *t*(111) = −3.315, *p* = 0.001, *d* = 0.724; IEDQOL: 1.65 ± 0.65 vs. 2.16 ± 0.48, *t*(110) = −3.817, *p* = 0.002, *d* = 0.893].

Table 1 shows the clinical presentation of the included sample divided by trauma history. The GLM analysis partially confirmed the presence of differences between EDE-Q total scores between subgroups controlling for age [*F*(3,111) = 0.3.201, *p* = 0.027], BMI [*F*(3,111) = 1.811, *p* = 0.151], diagnosis [*F*(3,111) = 0.2.074, *p* = 0.109], and their interaction [*F*(4,111) = 0.2.684, *p* = 0.036]. Also CIA results were partially confirmed controlling for age [*F*(3,112) = 4.432, *p* = 0.006], BMI [*F*(3,112) = 0.530, *p* = 0.663], diagnosis [*F*(3,112) = 3.926, *p* = 0.011], and their interaction [*F*(4,112) = 4.031, *p* = 0.005]. Differently, EDQOL showed no significant differences between subgroups controlling for age [*F*(3,82) = 1.850, *p* = 0.147], BMI [*F*(3,82) = 0.744, *p* = 0.530], diagnosis [*F*(3,82) = 0.796, *p* = 0.501], and their interaction [*F*(4,82) = 1.754, *p* = 0.150].

**Table 1:** Clinical presentation of the sample.

	nT	aT	cT	bT	F	*p* *η*^2^_p_ GLM*p*	*Post hoc*
	*n* = 27	*n* = 22	*n* = 23	*n* = 43			
Age, years	23.74 (9.94)	21.27 (3.44)	26.26 (9.22)	30.60 (11.81)	5.311	**0.002** 0.127	nT < bT (0.031) aT < bT (0.002)
BMI, kg/m^2^	16.74 (3.68)	16.13 (2.89)	17.30 (4.80)	22.41 (8.88)	7.467	**<0.001** 0.169	nT < bT (0.002) aT < bT (0.001) cT < bT (0.012)
SCL-90R tot	131.26 (50.37)	149.95 (73.69)	180.83 (50.92)	179.05 (55.38)	4.984	**0.003** 0.122 **0.001**	nT < cT (0.017) nT < bT (0.006)
EDE-Q restraint	3.11 (2.00)	4.16 (1.85)	4.14 (1.41)	3.74 (1.78)	1.856	0.141 0.049 0.219	
EDE-Q eating concern	2.95 (1.44)	3.30 (1.35)	3.59 (1.12)	3.92 (1.37)	3.058	**0.031** 0.078 0.079	nT < bT (0.025)
EDE-Q shape concern	4.01 (1.42)	4.88 (1.37)	5.56 (0.49)	5.11 (1.16)	8.048	**<0.001** 0.183 **0.005**	nT < cT (<0.001) nT < bT (0.001)
EDE-Q weight concern	3.39 (1.64)	4.57 (1.50)	4.96 (0.70)	4.50 (1.39)	6.290	**0.001** 0.149 **0.018**	nT < aT (0.030) nT < cT (0.001) nT < bT (0.007)
EDE-Q tot	3.37 (1.32)	4.23 (1.31)	4.56 (0.68)	4.32 (1.14)	5.570	**0.001** 0.134 **0.018**	nT < cT (0.002) nT < bT (0.006)
CIA	28.70 (9.88)	34.55 (9.26)	37.35 (6.40)	35.37 (10.94)	3.986	**0.010** 0.098 **0.025**	nT < cT (0.012) nT < bT (0.034)
IEDQOL tot	1.66 (0.65)	2.06 (0.38)	2.17 (0.51)	2.20 (0.51)	4.994	**0.003** 0.159 **0.001**	nT < cT (0.033) nT < bT (0.003)
CTQ tot	30.96 (4.75)	33.18 (4.64)	50.13 (12.75)	58.53 (16.90)	37.507	<0.001 0.503 <0.001	nT < cT (<0.001) nT < bT (<0.001) aT < cT (<0.001) aT < bT (<0.001)

BMI, body mass index; SCL-90R, Symptom Checklist-90-Revised; EDE-Q, eating disorder examination questionnaire; CIA, clinical impairment assessment; IEDQOL, Italian eating disorder quality of life; CTQ, childhood trauma questionnaire; nT, no trauma; aT, adult trauma; cT, child trauma; bT, both trauma; F = ANOVA with Bonferroni-corrected *post hoc* tests, results were considered significant for *p* < 0.05; *η*^2^_p_, partial eta-squared; GLM*p* = significance of the ANCOVA with age, diagnosis, and BMI as covariates. Significant *p*-values are reported with bold characters.

As for trauma symptomatology, the data indicate that trauma+ patients had more severe symptomatology than the trauma− subgroup for all the subscales of the TSI [A: 21.30 ± 6.11 vs. 17.56 ± 5.76, *t*(113) = −2.820, *p* = 0.006, *d* = 0.630; D: 17.64 ± 4.74 vs. 13.63 ± 5.34, *t*(113) = −3.728, *p* < 0.001, *d* = 0.200; AI: 14.10 ± 6.33 vs. 10.78 ± 5.51, *t*(113) = −2.456, *p* = 0.016, *d* = 0.559; IE: 14.16 ± 5.91 vs. 11.11 ± 5.21, *t*(113) = −2.406, *p* = 0.018, *d* = 0.547; DA: 12.89 ± 5.39 vs. 10.41 ± 4.43, *t*(113) = −2.173, *p* = 0.032, *d* = 0.503; DIS: 16.67 ± 5.73 vs. 13.74 ± 5.44, *t*(113) = −2.349, *p* = 0.020, *d* = 0.524; SC: 11.92 ± 6.04 vs. 8.22 ± 4.53, *t*(113) = −2.933, *p* = 0.004, *d* = 0.693; DSB: 9.31 ± 5.97 vs. 5.74 ± 4.59, *t*(113) = −2.854, *p* = 0.005, *d* = 0.670; ISR: 12.09 ± 5.81 vs. 6.48 ± 4.82, *t*(113) = −4.555, *p* < 0.001, *d* = 1.051; TRB: 11.56 ± 4.76 vs. 8.52 ± 3.86, *t*(113) = −3.023, *p* = 0.003, *d* = 0.701]. Regarding different trauma subgroups, data showed higher scores in bT group as reported in Table 2. Interestingly, there were significant differences between the aT group and bT group, with aT group scoring less than bT group.

**Table 2:** Trauma symptom inventory.

	nT	aT	cT	bT	F	*p* *η*^2^_p_ GLM*p*	*Post hoc*
AA	17.56 (5.76)	17.50 (7.10)	21.39 (6.44)	23.19 (6.21)	7.731	**<0.001** 0.173 **0.002**	nT < bT (0.001) aT < bT (0.001)
D	13.63 (5.34)	15.41 (3.89)	16.48 (4.76)	19.40 (4.54)	9.232	**<0.001** 0.200 **<0.001**	nT < bT (<0.001) aT < bT (0.009)
AI	10.78 (5.51)	12.18 (5.92)	13.22 (6.77)	15.56 (6.08)	3.789	**0.012** 0.093 **0.012**	nT < bT (0.010)
IE	11.11 (5.21)	12.18 (5.72)	13.09 (6.63)	15.74 (5.26)	4.328	**0.006** 0.105 **0.012**	nT < bT (0.007)
DA	10.41 (4.43)	11.64 (5.38)	12.35 (6.16)	13.81 (4.90)	2.561	0.059 0.065 0.118	
DIS	13.74 (5.44)	13.86 (5.13)	15.70 (5.81)	18.63 (5.78)	6.078	**0.001** 0.141 **0.018**	nT < bT (0.002) aT < bT (0.007)
SC	8.22 (4.53)	8.68 (5.24)	10.57 (5.68)	14.30 (5.72)	9.261	**<0.001** 0.200 **<0.001**	nT < bT (<0.001) aT < bT (0.001) cT < bT (0.049)
DSB	5.74 (4.59)	6.82 (3.87)	8.17 (5.62)	11.19 (6.49)	6.456	**<0.001** 0.149 **<0.001**	nT < bT (0.001) aT < bT (0.017)
ISR	6.48 (4.82)	10.41 (5.05)	10.52 (5.17)	13.79 (6.12)	10.024	**<0.001** 0.213 **<0.001**	nT < bT (<0.001)
TRB	8.52 (3.86)	10.09 (4.47)	10.39 (4.26)	12.93 (4.86)	5.895	**0.001** 0.137 **0.001**	nT < bT (0.001)
Trauma	39.07 (13.86)	41.32 (16.49)	46.83 (11.79)	52.74 (11.79)	5.981	**0.001** 0.139 **0.005**	nT < bT (0.001) aT < bT (0.019)
Dysphoria-self	70.70 (27.06)	79.45 (28.19)	87.70 (28.56)	103.49 (30.04)	8.928	**<0.001** 0.194 **<0.001**	nT < bT (<0.001) aT < bT (0.007)
Sex problems	22.48 (11.38)	25.59 (11.77)	29.13 (13.64)	38.42 (15.09)	9.294	**<0.001** 0.201 **<0.001**	nT < bT (<0.001) aT < bT (0.002) cT < bT (0.050)

nT, no trauma; cT, child trauma; bT, both trauma; *η*^2^_p_, partial eta-squared; AA, anxious arousal; D, depression; AI, anger/irritability; IE, intrusive experiences; DA, defensive avoidance; DIS, dissociation; SC, sexual concerns; DSB, dysfunctional sexual behavior; ISR, impaired self-reference; TRB, tension-reduction behavior; F = ANOVA with Bonferroni-corrected *post hoc* tests, results were considered significant for *p* < 0.05; GLM*p* = significance of the ANCOVA with age, diagnosis, and BMI as covariates. Significant *p*-values are reported with bold characters.

Clinical features of the personality traits and EMS of ED patients were also evaluated. In comparisons of the trauma+ and trauma− subgroups, our data showed significantly greater presence of EMS in the trauma+ [EMS-DR: 1.82 ± 1.09 vs. 1.10 ± 0.86, *t*(113) = 3.122, *p* = 0.002, *d* = 0.733; EMS-IAP: 2.00 ± 1.09 vs. 1.19 ± 0.89, *t*(113) = 3.507, *p* = 0.001, *d* = 0.814; EMS-ERS: 1.48 ± 1.09 vs. 0.95 ± 0.71, *t*(113) = 2.391, *p* = 0.018, *d* = 0.576; EMS-IL: 1.93 ± 1.16 vs. 1.07 ± 0.95, *t*(113) = 3.495 *p* = 0.001, *d* = 0.811]. Personality traits showed no significant differences due to the presence of any trauma. As for the different kinds of trauma, specific differences emerged between subgroups as showed in Table 3.

**Table 3:** Personality traits and early maladaptive schemas (EMS) domains.

	nT	aT	cT	bT	F	*p* *η*^2^_p_ GLM*p*	*Post hoc*
TCI-NS	51.15 (10.36)	45.23 (8.51)	47.74 (5.52)	59.56 (7.45)	19.696	**<0.001** 0.349 **<0.001**	nT < bT (<0.001) aT < bT (<0.001) cT < bT (<0.001)
TCI-HA	70.73 (13.33)	77.32 (8.99)	79.09 (5.89)	72.77 (11.92)	3.294	**0.023** 0.082 **0.050**	nT < cT (0.048)
TCI-RD	65.23 (10.80)	60.18 (7.68)	59.87 (6.75)	62.93 (7.67)	2.282	0.083 0.059 0.120	
TCI-P	62.38 (14.67)	69.59 (13.59)	62.78 (13.57)	62.35 (12.65)	1.655	0.181 0.043 0.627	
TCI-SD	59.77 (11.91)	52.59 (10.46)	49.91 (9.25)	51.49 (10.99)	4.306	**0.007** 0.105 **0.001**	cT < nT (0.011) bT < nT (0.015)
TCI-C	77.85 (8.11)	77.23 (11.29)	74.30 (5.29)	73.16 (7.16)	2.425	0.070 0.062 0.284	
TCI-ST	31.65 (9.23)	28.68 (7.29)	31.00 (7.82)	35.91 (9.62)	3.850	**0.012** 0.095 **0.005**	aT < bT (0.013)
EMS-DR	1.11 (0.86)	1.63 (1.08)	2.07 (1.25)	1.79 (1.01)	3.962	**0.010** 0.097 **0.006**	nT < cT (0.009)
EMS-IAP	1.19 (0.89)	1.92 (1.13)	2.23 (1.11)	1.91 (1.07)	4.576	**0.005** 0.110 **0.005**	nT < cT (0.004) nT < bT (0.035)
EMS-ERS	0.95 (0.71)	1.53 (1.01)	1.22 (0.97)	1.60 (1.84)	2.657	0.052 0.067 0.053	
EMS-IL	1.07 (0.95)	1.98 (0.96)	1.98 (1.23)	1.88 (1.24)	4.060	**0.009** 0.101 **0.006**	nT < cT (0.034) nT < aT (0.042) nT < bT (0.024)

TCI, temperamental and character inventory; NS, novelty seeking; HA, harm avoidance; RD, reward dependance; P, persistence; SD, self-directedness; C, cooperativeness; ST, self-transcendence; EMS, early maladaptive schema; EMS-DR, disconnection and rejection; EMS-IAP, impaired autonomy and performance; EMS-ERS, excessive responsibility and standards; EMS-IL, impaired limits; nT, no trauma; cT, child trauma; bT, both trauma; *η*^2^_p_, partial eta-squared; F = ANOVA with Bonferroni-corrected *post hoc* tests, results were considered significant for *p* < 0.05; GLM*p* = significance of the ANCOVA with age, diagnosis, and BMI as covariates. Significant *p*-values are reported with bold characters.

### Mediation Analysis

**Figure 1:**
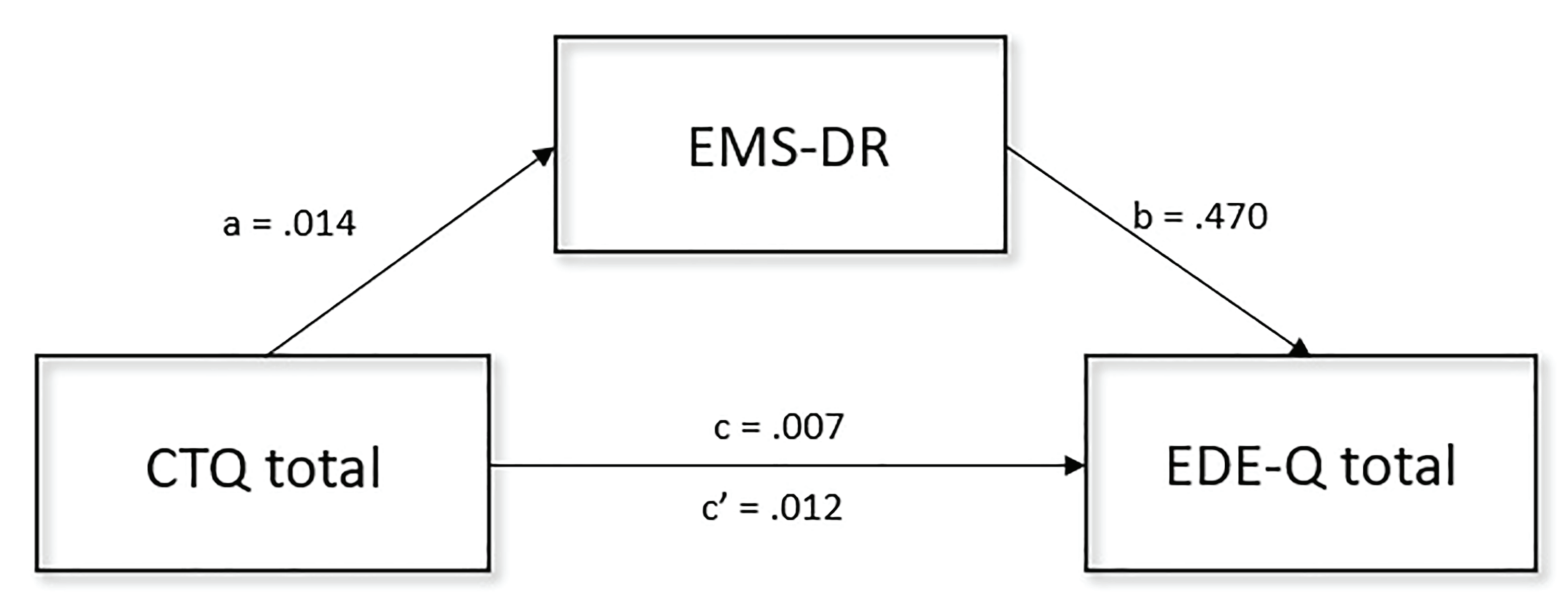
Graphical representation of the mediation analysis performed between CTQ total score (independent variable), EDE-Q total score (dependent variable), and EMS-DR (mediator). Confirmatory Sobel test confirmed the mediation effect represented (*t* = 2.152, SE = 0.003, *p* = 0.031). EDE-Q, eating disorder examination questionnaire; EMS-DR, early maladaptive schema disconnection and rejection; CTQ, childhood trauma questionnaire; a, b, c, and c' are path coefficients.

Different mediation analyses were performed to determine whether any personality traits or factors related to EMS had a mediator effect on the relationship between traumatic events exposure and eating psychopathology. Mediation analysis with all four EMS domains and all six TCI traits dides not result significantly with BTQ score as an independent variable. Looking at childhood trauma, the overall model results significant, but the only mediator that showed a significant effect was EMS-DR among all EMS and TCI variables [95% CI: EMS-IAP (−0.002, 0.017), EMS-ERS (−0.012, 0.003), EMS-IL (−0.001, 0.021), TCI-NS (−0.019, 0.006), TCI-HA (−0.003, 0.004), TCI-RD (−0.004, 0.003), TCI-P (−0.004, 0.004), TCI-SD (−0.001, 0.013), TCI-C (−0.001, 0.012), TCI-ST (−0.008, 0.002)]. The effect of childhood trauma on eating psychopathology was significantly mediated by EMS-DR [*β* = 0.007, SE = 0.003, 95% CI (0.002, 0.014)]. Furthermore, the CTQ score had a significant direct effect on EDE-Q [*β* = 0.012, SE = 0.304, 95% CI (0.001, 0.024)], childhood trauma had a direct effect on the EMS-DR score [*β* = 0.014, SE = 0.006, 95% CI (0.002, 0.026)], and EMS-DR had a direct effect on eating psychopathology levels [*β* = 0.470, SE = 0.095, 95% CI (0.282, 0.658)]. The overall proportion of the indirect effect was 35%. The mediation analysis is shown in Figure 1.

## Discussion

This paper’s main goal was to evaluate the differences in the psychopathological and psycho-functional profiles of ED patients with and without exposure to traumatic events at admission into a specialized inpatient ward. Different exposure times were considered to evaluate the difference among childhood, adulthood, or both childhood and adulthood exposures to traumatic events. The second goal was to evaluate possible psychological factors that could act as mediators of the effect of childhood trauma on the clinical severity of ED psychopathology.

From a clinical perspective, the patients who experienced traumatic events in this study had a more impaired psychopathological profily than the others, reportingmore severe ED psychopathology and psychological symptoms and a poorer quality of life. This evidence is in line with the recent literature from various different countries that has revealed a more complex profile in patients with traumatic experiences and various EDs (Backholm et al., 2013; Brewerton et al., 2020; Longo et al., 2021), and it corroborates the evidence of a specific adverse effect of trauma with a possible specific ecophenotype for ED patients with traumatic experiences (Chami et al., 2019). We confirmed the presence of a poorer quality of life and an impaired psychosocial domain (Backholm et al., 2013; Brewerton et al., 2020) in ED patients with traumatic events, supporting the importance of evaluating traumatic history in ED patients (Brewerton, 2019). Our data showed no significant differences between trauma subgroups (aT, cT, and bT) regarding psychopathology. Patients with traumatic experiences during childhood showed higher levels of specific psychopathology, more clinical impairment, and lower quality of life. Looking at specific trauma symptoms, significant differences raised between aT and other trauma-subgroups. Indeed, patients with only trauma exposures during adulthood reported fewer trauma symptoms than patients with also childhood trauma. This remark is in line with the idea that traumatic events have an impairment effect on individuals’ developmental life trajectories (Agorastos et al., 2019), extending the opinion that vulnerability is linked to childhood and early adulthood. Trauma could have a larger effect when people cannot rely on fully-developed psychological defenses (Waldinger et al., 2006) and its effect could also be amplified if it is recurrent or repeated (Clemmons et al., 2007; Cloitre et al., 2009). Our data partially corroborates this view because we have only quantified the trauma exposure and have not evaluated its quality. However, patients with cumulative trauma showed a more impaired clinical profile with higher psychopathology scores and higher physical symptomatology levels (Lin et al., 2016). This clinical profile could be linked to inflammation that has been a potential mediator between different trauma exposure and physical and mental well-being; in fact, people who reported a cumulative trauma exposure showed higher inflammation levels (Lin et al., 2016).

From a psychological perspective, our data indicated that in EDs patients an association between traumatic events and the EMS could be highlighted. In our study, patients with traumatic life-events showed higher EMSs without any differences attributable to the timing of exposure. The literature has already shown EMS’s potential role in the development and maintenance of psychopathology (Karatzias et al., 2016) and ED behaviors (Pugh, 2015). However, the ED’s EMS literature is still preliminary (Pugh, 2015), even though cognitive-behavioral approaches are considered the gold standard of treatments. Our data corroborate the evidence of a relationship between EMS and trauma (Pilkington et al., 2020), suggesting, however, that the time of trauma exposure does not influence them, even if the presence of the larger span between both cT and bT with respect to nT could be considered as an indicator for further studies, with larger samples, that could evaluate the relationship between childhood trauma and EMS, as already stated in general and clinical populations (Meyer et al., 2001; Messman-Moore and Coates, 2007). As for personality traits, our data provided weak evidence of differences between patients with and without trauma experiences, while the literature on healthy subjects has shown a robust connection between dysfunctional personalities and trauma (Carvalho et al., 2015). This evidence could confirm that personality is an essential aspect of the ED patients’ profiles (Wonderlich and Mitchell, 2001; Lilenfeld et al., 2006) and that personality could be only partially negatively affected by traumatic life events.

Finally, the mediation analysis showed a potential role for EMS in modifying the effect that early trauma has on eating psychopathology. This relationship has already been evaluated (Kong and Bernstein, 2009), however, the role of specific cognitive schemas as maintenance factors of specific psychopathology is still unclear. Specifically, our data showed the mediation role of the DR domain in the relationship between childhood trauma and eating psychopathology. However, it has to be noted that the direct effect of EMS-DR on EDE-Q was more significant than the indirect effect, showing the independent contribution that the domain “disconnection and rejection” has to eating psychopathology. Indeed, this domain is associated with attachment bonds and higher scores are linked to insecure attachment and the inability to form a secure connection, and these difficulties in the relationship with others were already largely reported in the ED literature, showing the perpetuation role of dysfunctional core beliefs exert (Jones et al., 2007; De Paoli et al., 2017). Our data adds to the evidence about the relationships between cognitive schema and eating psychopathology using a clinical sample and corroborating the idea that abandonment, mistrust/abuse, emotional deprivation, social isolation, and defectiveness could be dysfunctional coping strategies that characterized ED patients and that might be taken under consideration, especially in people with traumatic history (Sarin and Abela, 2003; Dakanalis et al., 2014; Pauwels et al., 2018; Meneguzzo et al., 2020a).

## Clinical Implication

The results of the present study suggest that traumatic experiences should be investigated in ED patients, especially when the severity of the psychopathology required hospitalization or led to the failure of different outpatient treatments. Indeed, traumatic experiences are associated with more severe ED psychopathology and more severe physical symptomatology. The presence of traumatic events should also be considered during the ED treatment, especially for the possible presence of cognitive schemas that should be taken into consideration. The role of trauma in cognitive schemas and personality traits requires to be investigated in further studies with different methodologies. However, our data showed the relevant presence of worse psychological profiles in patients with trauma exposure, especially in the interpersonal domains. Interpersonal schemas have indicated a significant association with eating psychopathology, supporting the interpersonal model for eating disorders and suggesting possible treatment approaches (Ivanova et al., 2015).

Moreover, core beliefs could be changed with specific treatment approaches, obtaining an improvement of the symptomatology with a personalized treatment approach (De Paoli et al., 2017). Finally, Scharff et al. (2021) reported that ED patients with a history of trauma could benefit from treatments focused on improving emotional functioning, including third-wave treatments. These treatments could help to improve the poor treatment outcome that standard cognitive behavior therapy has in ED patients with trauma, but more studies are needed (Castellini et al., 2018; Ke and Barlas, 2020; Serra et al., 2020). Our data add that specific cognitive schema should be treated in patients with early traumatic experiences, focusing on abandonment, mistrust/abuse, emotional deprivation, social isolation, and defectiveness.

## Limits and Conclusion

Some limitations of the present study need to be highlighted. Firstly, the study’s cross-sectional nature and the small samples size should be considered: it is not possible to evaluate any causal relationships among variables, and future studies should be longitudinal with bigger samples to replicate results and to evaluate interventions. Secondly, this study mostly relied on self-reported measures, which are open to bias. Thirdly, no control group was included due to the clinical origin of the data, and future studies should compare ED patients with healthy peers regarding the role of traumas in behaviors, thoughts, and specific concerns. Fourthly, our results’ generalization is limited by our sample’s characteristics: the unbalanced presence of men (5% of the sample), the recruitment of inpatient subjects of a rehabilitation facility and with drug therapy, the absence of a structured evaluation of the intelligence quotients, and severe ED psychopathology. Drug therapy was composed of SSRI, second generation of antipsychotics, and/or mood stabilizers, but none of the treatments used affected the participants’ ability to answer self-report questionnaires.

Despite these limitations, the current study highlights the relevance of traumatic events in the clinical presentation of ED patients. Trauma evaluation should be part of ED patients’ assessment and should be included in specific treatment protocols focused on specific cognitive maladaptive schemas. More studies are needed to identify the treatment outcome of patients with trauma exposure, and these studies should measure the effectiveness of addressing possible targets.

## Data Availability Statement

The raw data supporting the conclusions of this article will be made available by the authors upon motivated request.

## Ethics Statement

The studies involving human participants were reviewed and approved by the Internal Committee of the Casa di Cura Villa Margherita. Written informed consent to participate in this study was provided by the participants’ legal guardian/next of kin.

## Author Contributions

PM and PT have equally contributed to the structure, theoretical position, data analysis, and led manuscript writing. All authors have equally contributed to the data collection. All authors contributed to the article and approved the submitted version.

### Conflict of Interest

The authors declare that the research was conducted in the absence of any commercial or financial relationships that could be construed as a potential conflict of interest.
